# Report of a Novel ALOX12B Mutation in Self-Improving Collodion Ichthyosis with an Overview of the Genetic Background of the Collodion Baby Phenotype

**DOI:** 10.3390/life11070624

**Published:** 2021-06-27

**Authors:** Pálma Anker, Norbert Kiss, István Kocsis, Éva Czemmel, Krisztina Becker, Sára Zakariás, Dóra Plázár, Klára Farkas, Balázs Mayer, Nikoletta Nagy, Márta Széll, Nándor Ács, Zsuzsanna Szalai, Márta Medvecz

**Affiliations:** 1 Department of Dermatology, Venereology and Dermatooncology, Semmelweis University, 1085 Budapest, Hungary; anker.palma@phd.semmelweis.hu (P.A.); kiss.norbert@med.semmelweis-univ.hu (N.K.); becker.krisztina@med.semmelweis-univ.hu (K.B.); zakarias.sara@phd.semmelweis.hu (S.Z.); plazar.dora@phd.semmelweis.hu (D.P.); farkas.klara@phd.semmelweis.hu (K.F.); mayer.balazs@med.semmelweis-univ.hu (B.M.); 2 Department of Obstetrics and Gynaecology, Semmelweis University, 1082 Budapest, Hungary; kocsis.istvan@med.semmelweis-univ.hu (I.K.); eva.czemmel@gmail.com (É.C.); acs.nandor@med.semmelweis-univ.hu (N.Á.); 3 MTA-SZTE Dermatological Research Group, 6720 Szeged, Hungary; nagy.nikoletta@med.u-szeged.hu (N.N.); szell.marta@med.u-szeged.hu (M.S.); 4 Department of Medical Genetics, University of Szeged, 6720 Szeged, Hungary; 5 Department of Dermatology, Heim Pál National Children’s Institute, 1089 Budapest, Hungary; szalai.zsuzsannadr@gmail.com

**Keywords:** self-improving collodion ichthyosis, collodion baby, collodion membrane, ALOX12B, mutation, genodermatosis, disorder of cornification, genotype, autosomal recessive congenital ichthyosis

## Abstract

Collodion baby is a congenital, transient phenotype encountered in approximately 70–90% of autosomal recessive congenital ichthyosis and is an important entity of neonatal erythroderma. The clinical outcome after this severe condition is variable. Genetic mutations of components of the epidermal lipoxygenase pathway have been implicated in the majority of self-improving collodion ichthyosis (SICI). In SICI, the shedding of the collodion membrane reveals clear skin or only mild residual manifestation of ichthyosis. Here we report the case of a girl born with a severe form of collodion baby phenotype, whose skin almost completely cleared within the first month of life. At the age of 3 years, only mild symptoms of a keratinization disorder remained. However, the severity of erythema and scaling showed mild fluctuations over time. To objectively evaluate the skin changes of the patient, we assessed the ichthyosis severity index. Upon sequencing of the ALOX12B gene, we identified a previously unreported heterozygous nonsense mutation, c.1607G>A (p.Trp536Ter) with the recurrent, heterozygous mutation c.1562A>G (p.Tyr521Cys). Thereby, our findings expand the genotypic spectrum of SICI. In addition, we summarize the spectrum of further genetic diseases that can present at birth as collodion baby, in particular the SICI.

## 1. Introduction

Autosomal recessive congenital ichthyosis (ARCI) is a major subgroup of the non-syndromic forms of congenital ichthyosis characterized by abnormal skin cornification with hyperkeratosis, diffuse scaling and variable degree of erythroderma. ARCI is a rare condition, the reported prevalence varies between 1:33,000 and 1:300,000 [1,2,3]. ARCI has diverse clinical manifestations. To date, the mutational spectrum encompasses ten genes that encode proteins (enzymes, transport proteins) responsible for the formation of the stratum corneum [4,5]. Subtypes of ARCI traditionally include lamellar ichthyosis (LI) with large, dark, plate-like scales without erythroderma, and congenital ichthyosiform erythroderma (CIE) with generalized fine scaling and erythroderma. Although LI and CIE are considered two distinct clinical entities, patients often show overlapping features [6]. The most severe subtype of ARCI is Harlequin ichthyosis (HI), which is triggered by inactivating mutations of the *ABCA12* gene [7,8]. About 70–90% of ARCI newborns are born encased in a parchment-like membrane, referred to as the collodion membrane [9,10]. It is noteworthy that the prevalence might be even higher, since due to the occasionally mild clinical presentation, most of the cases may go unreported [11]. The term collodion baby (CB) refers to an early, transient phenotype, most commonly encountered in the case of ARCI, rather than a distinct disease entity of ichthyoses. In the majority of cases, the shedding of the collodion membrane is followed by the development of LI or CIE. About 10–20% of CBs show clear skin or only mild symptoms of ichthyosis later on, which was previously referred to as self-healing collodion baby (SHCB). However, the term self-improving collodion ichthyosis (SICI) is preferable instead of SHCB as this minor group of ARCI patients usually still show residue symptoms of LI or CIE. Vahlquist et al. proposed the umbrella term pleomorphic ichthyosis for cases characterized by marked skin changes at birth and subsequently mild symptoms of ichthyosis encompassing SICI, ichthyosis prematurity syndrome, bathing-suit ichthyosis and congenital ichthyosis with mild scaling [4,12]. SICI is predominantly associated with mutations in the *ALOX12B*, *ALOXE3* and less often *TGM1* genes [1,13,14]. Lately, the mutation spectrum was expanded with the *CYP4F22* gene [9,14]. The severity of ichthyosis in later life in the case of CB is diverse, ranging from HI to variable degree of LI/CIE and SICI. While genetic testing is an important tool for the diagnosis of ichthyosis, the outcome of CB usually cannot be accurately predicted at birth [15]. Here, in addition to a brief review of the literature regarding the diversity of genotype-phenotype correlation of CB, we report a case of a SICI with a novel mutation of the *ALOX12B* gene that further expands the genotypic spectrum of SICI.

## 2. Materials and Methods

### 2.1. Disease Severity Assessment

We applied the disease severity score for newborns with collodion membrane of Rubio-Gomez et al. to describe the status of the newborn patient [15]. In addition, to objectively assessing the skin changes of the patient, we used the ichthyosis severity index of Marukian et al. [16]. Erythema and scaling were evaluated on the upper back region of the patient.

### 2.2. Mutation Analysis

Genomic DNA was isolated from peripheral blood leukocytes of the patient and her parents with a Roche MagNA Pure Compact system (Roche Diagnostics, Mannheim, Germany) or with a BioRobot EZ1 DSP Workstation (QIAGEN; Hilden, Germany), for *TGM1* or *ALOX12B* and *ALOXE3,* respectively. After the amplification of the coding regions and flanking introns of the *TGM1* (primer pairs for PCR were as described previously [17,18]), *ALOX12B* and *ALOXE3* genes (using primer sequences displayed on the UCSC Genome Browser, http://www.genome.ucsc.edu, accessed on 24 April 2019), DNA sequencing was performed on amplification products. Sequencing data were analyzed in order to screen for any genetic variations.

Written informed consent was obtained from the parents of the patient, and the study was conducted according to the Principles of the Declaration of Helsinki.

## 3. Results

### 3.1. Case Report

**Figure 1:**
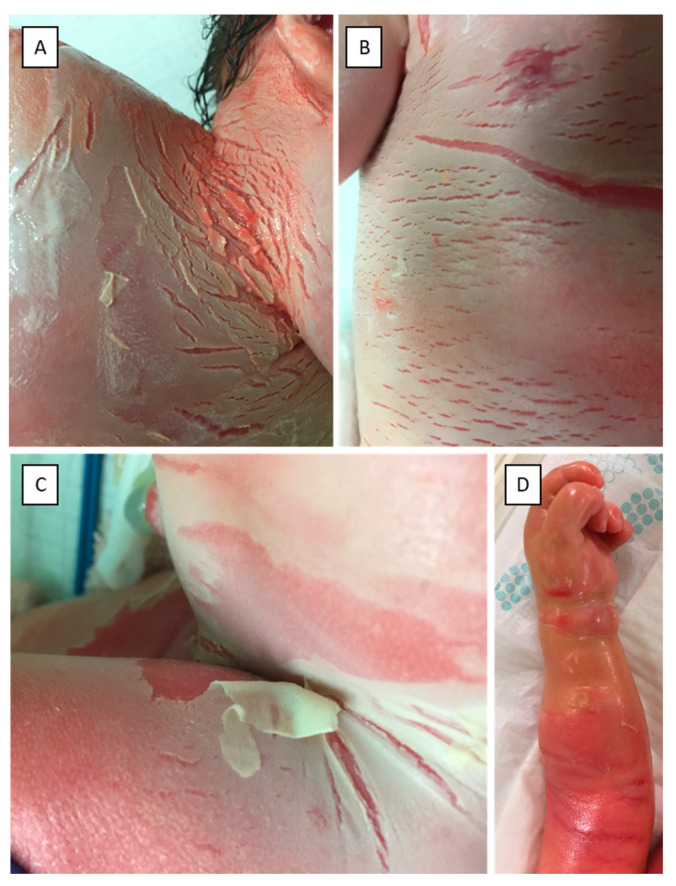
Clinical pictures of the neck (**A**), chest (**B**) and inguinal region (**C**) of the newborn patient. The newborn was covered in an opaque membrane with underlying erythroderma. The neck, trunk and inguinal region presented with several fissures. Picture (**D**) taken at 2 days of age shows compression by the tight collodion, which covers the edematous hand and the arm of the patient.

**Figure 2:**
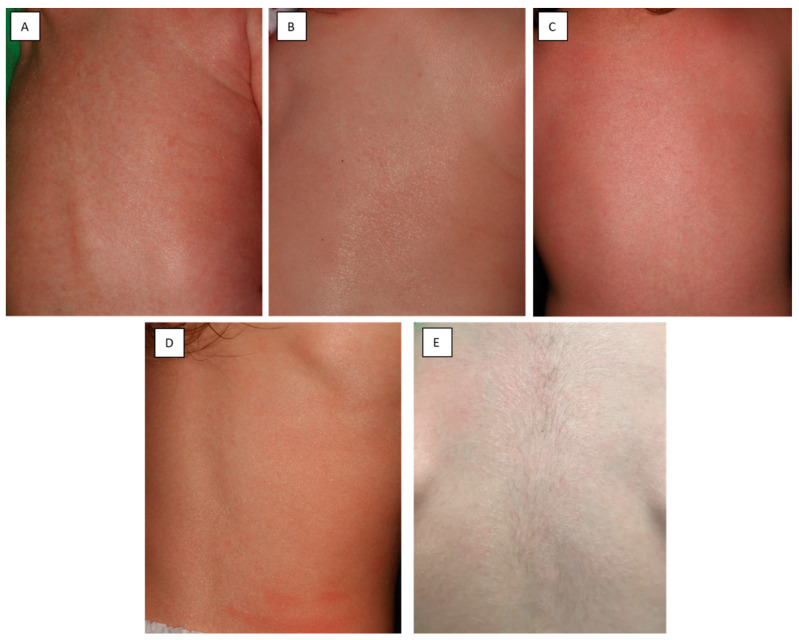
Clinical photographs of the back of the patient taken at 1 month (**A**), 3 months (**B**), 6 months (**C**), 1.5 years (**D**) and 3 years (**E**) of age. The severity of erythroderma fluctuated over time.

Here we report the case of a three-year-old girl, who was born to non-consanguineous parents at 37 gestational weeks following an uncomplicated pregnancy, through a normal vaginal delivery as a CB. The parents had no relevant history of skin diseases. The newborn was covered in an opaque membrane with underlying erythroderma (Figure 1A–C). The intertriginous regions and the trunk presented with several fissures. Marked ectropion and eclabium could be noted as well. Routine neonatal assessment was otherwise normal with an Apgar score of 9/9 at 1 and 5 min, respectively. Due to the marked skin changes, the newborn was transferred to a perinatal intensive care unit. The skin status dynamically changed during the first few days postpartum. Due to the compression of the shiny, tight collodion membrane, the extremities appeared edematous and fingers were fixed in a contracture (Figure 1D). Elevated inflammatory markers and positive skin and blood culture showed signs of a multimicrobial infection, which was treated successfully with combined intravenous antibiotic therapy. The newborn had severe anemia secondary to the infection, for which she received blood transfusion. Her skin improved following treatment with topical emollients; the ectropion and eclabium healed and she was emitted from the intensive care unit at the age of 18 days. By the age of one month, the shedding of the collodion membrane revealed erythroderma with fine white scaling. Over time, the severity of the erythema and scaling fluctuated (Figure 2). Currently, at age of three, the patient has mild residual manifestation of ichthyosis, namely xerosis and mild erythematous macules.

### 3.2. Disease Severity

**Figure 3:**
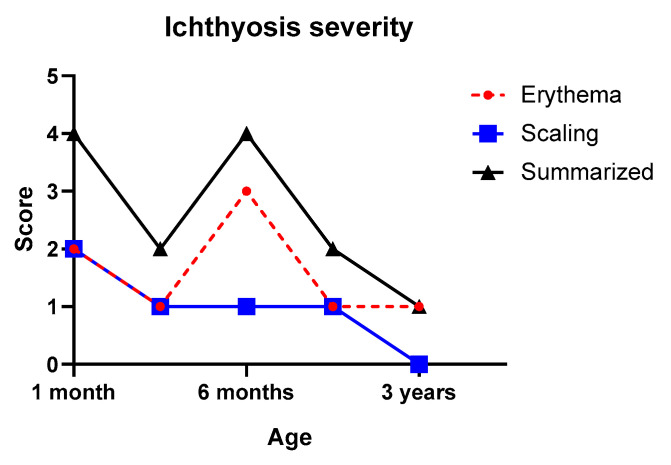
Changes of ichthyosis severity index of the patient. Erythema and scaling were evaluated on the upper back region of the patient.

Collodion membrane severity score was 12 points out of 15, which accounts for high severity. Changes of the ichthyosis severity index after the neonatal period, including the erythema and scaling score, are seen in Figure 3. Scaling was mild after 1 month of age, while the erythema score fluctuated during the observed time period.

### 3.3. Mutation Analysis

**Figure 4:**
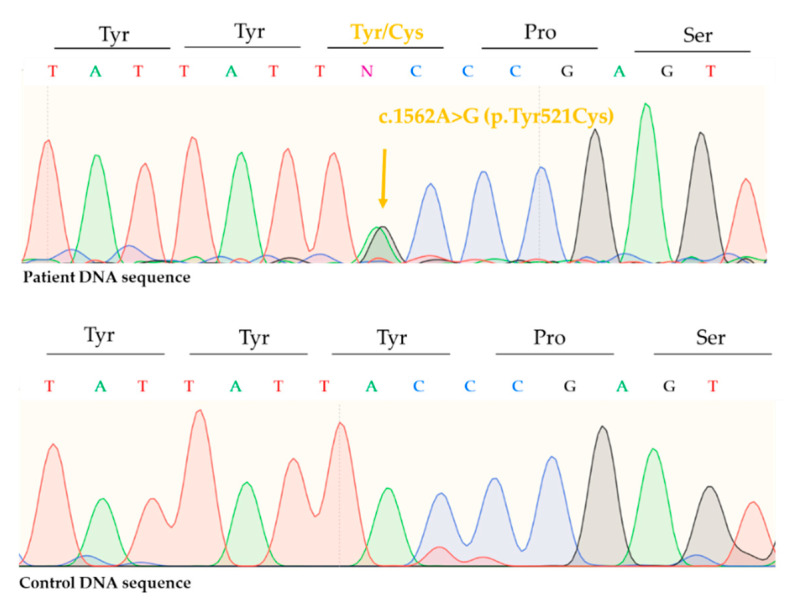
Direct sequencing revealed a heterozygous, recurrent, pathogenic, missense mutation on the *ALOX12B* gene c.1562A>G (p.Tyr521Cys).

**Figure 5:**
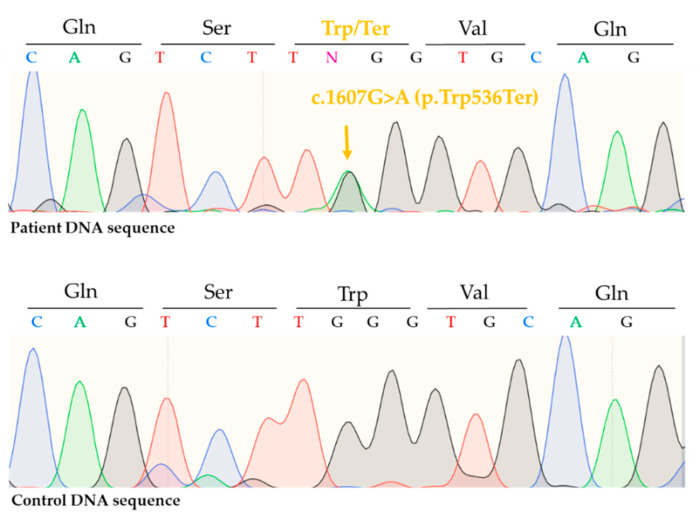
Direct sequencing revealed a novel, nonsense mutation on the *ALOX12B* gene in heterozygous form c.1607G>A (p.Trp536Ter).

Sequencing of *ALOX12B* revealed a previously described pathogenic, missense mutation, c.1562A>G (p.Tyr521Cys) in one allele (Figure 4) and a previously not reported, nonsense mutation c.1607G>A (p.Trp536Ter) in the other allele (Figure 5). Thus, the patient had a compound heterozygote mutation in the *ALOX12B* gene. 

Upon sequencing of *TGM1* and *ALOXE3*, wild type alleles were detected. Parental testing revealed c.1607G>A (p.Trp536Ter) heterozygous mutation of *ALOX12B* in the mother and c.1562A>G (p.Tyr521Cys) heterozygous mutation of *ALOX12B* in the paternal sample.

## 4. Discussion

ARCI is a genetically heterogeneous group of cornification disorders with diverse and often dynamically changing or overlapping phenotypes. The presence of collodion membrane at birth is often the initial presentation of ARCI. Later, CB develops into CIE/LI or, on rare occasions, heals spontaneously. This latter phenotype is known as SICI, where patients show clear or almost clear skin with mild signs of ichthyosis. 

Here we reported the case of a CB, whose symptoms improved significantly after birth. Sequencing of the *ALOX12B* gene revealed c.1526A>G (p.Tyr521Cys), a frequently reported, well-documented mutation and a novel, pathogenic nonsense mutation, c.1607G>A (p.Trp536Ter). Vahlquist et al. reported five SICI patients with the c.1526A>G (p.Tyr521Cys) mutation [19]. Before that, this particular mutation was only documented in case of other forms of ARCI (LI/CIE) [19]. In a new meta-analysis exploring the genotypic spectrum of *ALOX12B* and *ALOXE3* mutations, c.1526A>G (p.Tyr521Cys) was the most frequent mutation, with an allelic frequency of 22% (61 out of 282 alleles) [5]. Although c.1526A>G (p.Tyr521Cys) frequently occurs in SICI cases, it was detected in numerous LI and CIE patients as well, both in homozygote and compound heterozygote form [5]. Based on this meta-analysis, CB phenotype was documented in 25 out of 44 cases with either heterozygous or homozygous form of c.1526A>G (p.Tyr521Cys) mutation, including five SICI cases. On the other hand, to the best of our knowledge, the other mutation of the patient, c.1607G>A (p.Trp536Ter), has not yet been reported in the literature. The novel c.1607G>A (p.Trp536Ter) mutation affects exon 12 of *ALOXB12* gene and causes a premature stop codon.

Of note, in case of mutations of genes encoding the lipoxygenase enzymes, the outcome of CB cannot be accurately predicted neither based on genetic analysis nor the initial clinical presentation [5]. In addition, in our experience, the severity of the disease, e.g., the degree of erythema, can show relapses and remissions that probably are influenced by variable exogenous and endogenous factors. The background of the dramatic improvement of the skin status in SICI after birth is unknown. In the case of *TGM1* mutations, two mutated alleles were found to be sensitive to hydrostatic pressure that results in inactive transglutaminase-1 enzyme in utero, which explains the severe phenotype in the newborn that later resolves in normal environmental conditions [20]. Such an explanation does not exist in the case of the lipoxygenase pathway genes. However, the fact that patients with identical mutations can demonstrate different outcomes suggests the role of yet undiscovered factors. A multicentric study confirmed a similar trend as Rubio-Gomez et al.—toward a higher collodion membrane severity score in the case of non-syndromic forms of ichthyosis compared to syndromic forms [15,21]. However, this study could not establish a strong link between the collodion membrane score and the clinical outcome of the disease. The authors hypothesized that it was due to the fact that genes that cause non-syndromic forms of ichthyosis are related to a higher differentiation state of keratinocytes. Our patient had a high ichthyosis severity score, which fits in with the trend that these two studies established. However, the outcome of the SICI phenotype could not be predicted based on this scoring system. While collodion membrane at birth occurs most commonly in ARCI, it must be noted that other diseases can present at birth as CB. These include other forms of syndromic and non-syndromic forms of ichthyosis, hypohidrotic ectodermal dysplasia, palmoplantar keratosis with leukokeratosis anogenitalis [15], congenital hypothyroidism [22,23], alpha-ketoadipic aciduria [24,25] and koraxitrachitic syndrome [26] (Table 1 and Table 2).

It is important to note that while these diseases can present as CB at birth, most of them are rare exceptions in the literature. So far, two cases with congenital hypothyroidism and one with alpha-ketoadipic aciduria were reported to be born as CB [22,23,24]. Although koraxitrachitic syndrome is frequently cited in association to rare causes of the CB phenotype, there are very few publications about this rare entity [26]. A recent article reported the case of a Harlequin fetus-like newborn, who improved in the first weeks and presented with palmoplantar keratoderma and leukokeratosis anogenitalis caused by *KDSR* mutation. Autosomal recessive mutations of *KDSR* are the cause of erythrokeratodermia variabilis et progressiva 4 as well where a vernix-like thickened skin was documented at birth, in some cases besides a true collodion membrane [55]. Another interesting point is the difference of CB and Harlequin ichthyosis at birth. By definition, Harlequin fetus is a neonatal phenotype that later develops to the most severe form of ichthyosis. According to the First Ichthyosis Consensus Conference in Sorèze, the Harlequin fetus is a severe form of CB with thick plate-like, cornified skin [59]. However, due to the marked skin changes and different clinical consequences, it is often referred to as a distinct disease entity in the literature [10,60]. Additionally, it is important to note that there are neonatal conditions that can be confused with the CB phenotype, including ichthyosis prematurity syndrome (OMIM# 604194) and keratitis-ichthyosis-deafness syndrome (OMIM# 600157). The marked skin changes at birth resemble excessive amounts of vernix caseosa in these cases [61]. However, due to the lack of a unified nomenclature, some of these cases are reported as a collodion membrane in the literature [9].

## 5. Conclusions

In conclusion, we described the case of a SICI, where the patient had a compound heterozygous mutation in the *ALOX12B* gene. Here, we identified a previously unreported, pathogenic, nonsense mutation c.1607G>A (p.Trp536Ter) along with a recurrent, pathogenic, missense mutation, c.1562A>G, (p.Tyr521Cys). Our case supports the notion that the clinical interpretation and the determination of the prognosis based on genotype–phenotype correlation regarding SICI is challenging. The clinical outcome of a CB cannot always be accurately predicted based on the severity of the clinical presentation at birth; thus, genetic testing is essential. However, in the case of mutations of the lipoxygenase enzyme genes, an association between specific mutations and the mature phenotype could not yet be confirmed. Thus, counseling and education of the parents are of great importance in these cases.

## Figures and Tables

**Table 1:** Variable forms of ARCI as the most common cause of the collodion baby phenotype with the reported frequency in the literature.

Forms of ARCI	Gene(s)	OMIM	Frequency of the CB Phenotype	Reference No.
Harlequin ichthyosis	*ABCA12*		100%	[9]
Lamellar ichthyosis /Congenital ichthyosiform erythroderma	*TGM1*	242,300	73–97%	[9,27]
	*ALOX12B*	242,100	71–76%	[9]
	*ALOXE3*	606,545	36–45%	[9]
	*NIPAL4*	612,281	15–33%	[9,28]
	*CYP4F22*	604,777	50%	[29]
	*ABCA12*	601,277	100%	[9]
	*PNPLA1*	615,024	58–67%	[9,30,31]
	*CERS3*	615,023	100%	[9,32,33,34]
	*SDR9C7*	617,574	0–100%	[9,35,36]
	*SULT2B1*	617,571	67–100%	[37,38]
	locus 12p11.2-q13.1	615,022	0%	[39]
Self-improving collodion ichthyosis	*ALOX12B*	242,100	100% ^#^	[19]
	*ALOXE3*	606,545		[19]
	*TGM1*	242,300		[19]
	*CYP4F22*	604,777		[14]
Bathing-suit ichthyosis	*TGM1*	242,300	88–100%	[40]
Acral self-healing collodion baby	*TGM1*	242,300	100% ^#^	[41]

#: by definition; ARCI: autosomal recessive congenital ichthyosis.

**Table 2:** Rare causes of the collodion baby phenotype.

Disease	Mode of Inheritance	Gene(s)	OMIM	Reference No.
**Non-syndromic ichthyosis**				
*Common ichthyosis*				
Ichthyosis vulgaris	ASD	*FLG*	146,700	[42,43]
Recessive X-linked ichthyosis	XR	*STS*	308,100	[42,44]
*Other forms of ichthyosis*				
Loricrin keratoderma (Vohwinkel syndrome with ichthyosis)	AD	*LOR*	604,117	[45]
Congenital reticular ichthyosiform erythroderma/Ichtyosis variegata/Ichthyosis with confetti	AD	*KRT10*	609,165	[46,47]
**Syndromic Ichthyosis**				
Recessive X-linked ichthyosis, syndromic form	XR	*STS* (and others *)	308,100	[44]
IFAP-syndrome	XR	*MBTPS2*	308,205	[48]
Conradi–Hünermann–Happle syndrome (CDPX2)	XD	*EBP*	302,960	[42]
MEND syndrome	XR	*EBP*	300,960	[49]
Comel–Netherton syndrome	AR	*SPINK5*	256,500	[50]
Trichothiodystrophy (Tay syndrome)	AR	*ERCC2/XPD*	601,675	
		*ERCC3/XPB*	616,390	[50]
		*GTF2H5/TTDA*	616,395	
Sjögren–Larsson syndrome	AR	*ALD3A2*	270,200	[50]
Gaucher syndrome type 2	AR	*GBA*	230,900	[51,52]
Neutral lipid storage disease with ichthyosis (Chanarin–Dorfman syndrome)	AR	ABHD5	275,630	[50]
**Others**				
Hypohidrotic ectodermal dysplasia	XR	*EDA*	305,100	[53,54]
Ankyloblepharon–ectodermal dysplasia–cleft lip/palate syndrome (Hay–Wells syndrome)	AD	*TP73L*	106,260	[53]
Erythrokeratodermia variabilis et progressiva 4	AR	*KDSR*	617,526	[55]
Palmoplantar keratosis with leukokeratosis anogenitalis	AR	*KDSR*	n/a	[56,57]
Koraxitrachitic syndrome	n/a	n/a	n/a	[26]
α-ketoadipic aciduria	AR	*DHTKD1*	204,750	[24,25]
Holocarboxylase synthetase deficiency	AR	*HLCS*	253,270	[58]
Congenital hypothyroidism ^#^	AD	*PAX8*	218,700	
	AR	*TSHR*	275,200	
		*DUOX2*	607,200	
		*SLC5A5*	274,400	[22,23]
		*TG*	274,700	
		*TPO*	274,500	
		*TSHB*	275,100	

* in context of contiguous gene syndrome; # genetic causes account for 15–20% of the cases. Abbreviations: AD, autosomal dominant; ASD, autosomal semidominant; AR, autosomal recessive; XR, X-linked recessive; n/a: no data available; CDPX2, chondrodysplasia punctata type 2; MEND, male EBP disorder with neurological defects.

## Data Availability

The datasets generated during and/or analysed during the current study are available from the corresponding author on reasonable request.
